# Liver injury in COVID-19: The current evidence

**DOI:** 10.1177/2050640620924157

**Published:** 2020-05-26

**Authors:** Saleh A Alqahtani, Jörn M Schattenberg

**Affiliations:** 1 Liver Transplantation Unit, King Faisal Specialist Hospital & Research Center, Riyadh, Saudi Arabia; 2 Division of Gastroenterology and Hepatology, Johns Hopkins University, Baltimore, USA; 3 Metabolic Liver Research Program, I. Department of Medicine, University Medical Center, Mainz, Germany

**Keywords:** SARS-CoV2, COVID-19, liver injury, liver function test, cholangiocytes, lymphopenia, cytokine storm

## Abstract

Patients with novel coronavirus disease 2019 (COVID-19) experience various degrees of liver function abnormalities. Liver injury requires extensive work-up and continuous surveillance and can be multifactorial and heterogeneous in nature. In the context of COVID-19, clinicians will have to determine whether liver injury is related to an underlying liver disease, drugs used for the treatment of COVID-19, direct effect of the virus, or a complicated disease course. Recent studies proposed several theories on potential mechanisms of liver injury in these patients. This review summarizes current evidence related to hepatobiliary complications in COVID-19, provides an overview of the available case series and critically elucidates the proposed mechanisms and provides recommendations for clinicians.

## Key points

- Altered liver function tests are reported in up to half of the patients with COVID-19 infection.
- Disease severity, pre-existing liver disease and older age present a risk for liver injury.
- Drug-induced liver injury is an important consideration in patients with COVID-19.
- Hepatotoxic antiviral medications require careful monitoring of adverse effects.
- SARS-CoV-2 may directly bind to ACE2 positive cholangiocytes and can cause hepatic injury.
- Activation of the immune system and ‘cytokine storm’ may contribute to an immune-mediated process of hepatic injury in COVID-19.
- The control of cytokine dysregulation at an early stage could be beneficial to curb the disease progression.

## Introduction

In the current pandemic coronavirus disease (COVID-19), almost every country in the world has now registered COVID-19 cases, and the confirmed cases have exceeded one million to date. While initial clinical studies, especially from China, the USA and Italy, have highlighted the dominant clinical symptoms including fever, cough, fatigue and shortness of breath, the later research unveiled shreds of evidence on the extrapulmonary manifestations of the disease. These reports highlighted that beyond severe acute respiratory syndrome coronavirus 2 (SARS-CoV-2), a complicated course of the disease or even viral infection itself can lead to involvement of other organs and multi-organ failure. The liver is the primary organ for detoxification and metabolism, and maintaining an optimal function is imperative to engage all available therapeutic modalities in the treatment of COVID-19. Abnormal liver function requires clinical evaluation, continuous surveillance and, potentially, specific therapy. To support clinical decision making and optimize the outcome in the treatment of COVID-19, it will be crucial to clearly understand the possible mechanisms involved in liver injury. The current review summarizes the pathophysiology and potentially specific role of COVID-19 in liver disease based on the available data and case series published, ahead of print and non-peer-reviewed preprints as of 2 April. The search strategy is detailed in the Supplementary Material online.

## Pathophysiological basis of liver injury in patients with COVID-19

**Figure 1.**
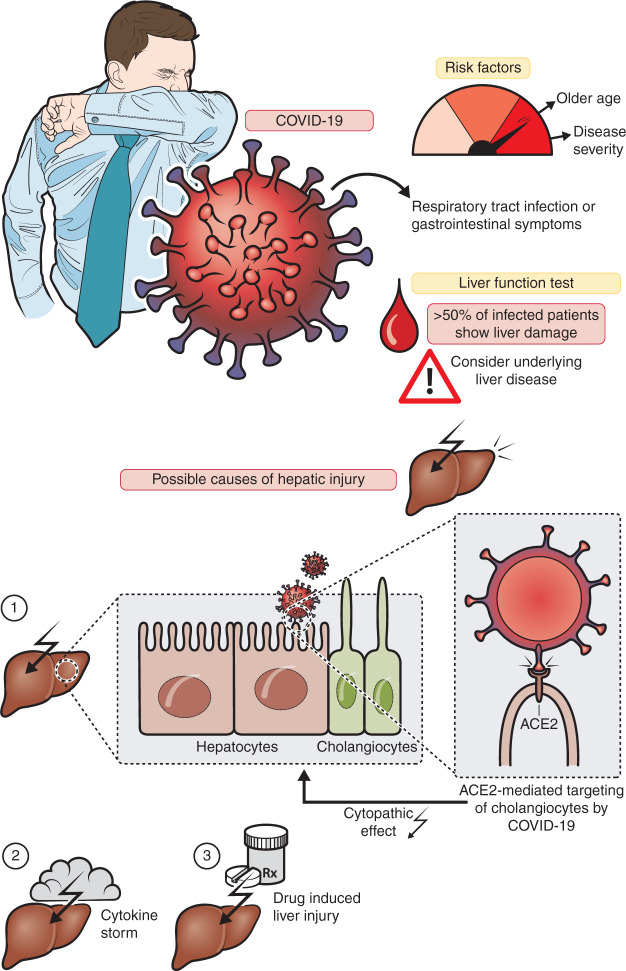
Clinical characteristics and pathophysiology of liver injury from COVID-19. ACE2: angiotensin-2 converting enzyme

Emerging data from small clinical case studies have proposed that liver injury in COVID-19 is frequently seen, but the extent and underlying mechanisms remain undetermined. Figure 1 summarizes the pathophysiological findings, which are discussed below.

### Direct viral effect on the liver

The liver exerts a crucial function in host defense against microbes and is involved in most systemic infections as it receives both the portal and systemic circulation. Certain viruses exert a direct cytopathic effect on hepatocytes and cholangiocytes although, in most cases, the pathogenesis seems multifactorial. Yang et al. reported that SARS-CoV could cause direct cytopathic liver injury rather than inducing cellular stress from low oxygen supplies or cytokines as seen in sepsis.^1^ Autopsy studies in patients revealed that SARS-CoV was detectable in 41% of the liver tissue, with a maximum viral load of 1.6 × 10^6^ copies/g of tissue.^2^ The pathological findings of liver biopsy specimens from SARS patients showed hepatocellular necrosis, mitoses, cellular infiltration and fatty degeneration. In a recent autopsy analysis of liver tissue from a patient with COVID-19, moderate microvesicular steatosis and mild inflammation in the lobular and portal area was observed. However, this pattern of histological injury is not specific for one etiology but can also be observed during sepsis or drug-induced liver injury (DILI).^3^

### The role of cholangiocytes in COVID-19

Similar to SARS-CoV, SARS-CoV-2 uses the angiotensin-2 converting enzyme (ACE2) receptor protein to attack the host system.^4^ The cell entry receptor, ACE2, is widely expressed across the human body, including the lungs (type II alveolar cells), gastrointestinal tract (esophageal epithelial cells and absorptive enterocytes of ileum and colon), hepatobiliary system (hepatocytes and cholangiocytes), cardiovascular system (myocardial cells), the renal system (proximal tubule cells and urothelial bladder cells) and the pancreas.^5^ Recent studies have observed that ACE2 expression in the cell clusters of cholangiocytes was significantly higher than that in the hepatocytes population (59.7% *vs*. 2.6%).^6^ The authors conclude that SARS-CoV-2 may directly bind to ACE2 positive cholangiocytes, but not hepatocytes, to exert a cytopathic effect. Cholangiocytes are involved in many aspects of liver physiology, including regeneration and adaptive immune response mechanisms, and the disruption of cholangiocyte function can cause hepatobiliary damage. This is supported by cholestatic markers, including gamma-glutamyl transferase (GGT), that can be found in some, but not all, case series of COVID-19.^7–9^ Notably, a recent review reported unpublished data with GGT elevations in 54% of cases.^8^ In a human organoid model of liver ductal organoids, permissiveness to SARS-CoV-2 infection was observed. Here viral infection impaired the barrier and bile acid transporting functions of cholangiocytes through dysregulation of genes involved in tight junction formation and bile acid transportation, supporting the susceptibility of cholangiocytes in SARS-CoV-2-related liver injury.^10^

### Activation of the immune system in COVID-19

Dysregulation of the innate immune response can be one aspect of liver injury in COVID-19. Patients with COVID-19 exhibit marked activation of inflammatory markers, including abnormal levels of C-reactive protein (CRP), lymphocytes, neutrophils and cytokines, in particular interleukin-6 (IL-6).^8^,^11–13^ These mechanisms may contribute to pulmonary and extrapulmonary injuries^12^,^14^ and the control of cytokine dysregulation at an early stage could be beneficial to curb the disease progression.^15^

Hepatic inflammation involving activation of innate immune cells and the release of cytokines is a well-established driver of liver injury from various causes.^16^ In some of the available case series of COVID-19, a correlation between lymphopenia and liver injury was observed and CRP ≥20 mg/L and a lymphocyte count <1.1 × 10^9^/L were independent risk factors for liver injury. Notably, lymphopenia in COVID-19 studies was reportedly observed in 63% to 70.3% of patients and those with lower lymphocyte counts more susceptible to fatal outcomes.^11^

## Clinical evidence

### Elevated liver function tests (LFTs) in COVID-19

More than 20 publications to date reported abnormal levels of aminotransferases in patients with COVID-19.^7^^--^^9^,^11–13^,^17--19,21,23--27,29–33,35,36^ A recent systematic review and meta-analysis on LFT abnormalities provided a pooled elevation of aspartate aminotransferase (AST) in 33.3% and alanine aminotransferase (ALT) in 24.1% of cases.^39^ Various investigators across different studies reported a correlation between the severity of COVID-19 and the degree of liver dysfunction.^8^,^11^,^25^ In one retrospective study, one patient experienced severe hepatitis with ALT of 7590 U/L and AST of 1445 U/L.^17^ In a report from Shanghai, 50.7% of patients presented with elevated LFTs at the time of hospitalization. Interestingly, these were more likely to have a moderate-to-high-grade fever when compared with the patients with normal LFT (44% *vs*. 27.4%; *p* = 0.035).^7^ On the other hand, mild and moderate cases experienced only discrete abnormal LFT values. These reports support the concept that the disease severity and an older age predispose to more severe liver injury from COVID-19. Based on these case series, patients with severe COVID-19 and pre-existing liver conditions^8^ – but also elderly patients^11^ – should undergo surveillance and individually tailored therapeutic approaches for potential liver injury.

A recently published article by Bangash et al. argues after careful review of seven relevant studies that elevated ALT and AST may not necessarily be of hepatic origin alone. The authors have given a timely reminder that it is common for other respiratory viruses to create similar LFT elevations^28^ and thus more prospective data related to the clinical relevance COVID-19 and liver injury is required.

The previous pathogenic coronaviruses, such as SARS-CoV and the Middle East respiratory syndrome coronavirus (MERS-CoV), were also reported to manifest with elevated levels of ALT and AST.^39^ More generally, non-hepatotropic viral infections may affect the liver and induce hepatitis or fulminant acute liver failure. However, in the majority of cases, recovery from viral illness is often sufficient to resolve liver injury.^40^

Like in SARS-CoV and MERS-CoV infections, abnormal levels of albumin and lactate dehydrogenase (LDH) were also reported in SARS-CoV-2 infection, with the maximum of 98% and 76% of the patients affected as reported in the study by Chen et al.^17^ It is important to remember that LDH and AST elevation could be from muscle damage and not necessarily reflect liver injury.

### DILI

The current armory of therapeutic agents explored against SARS-CoV-2 includes several antiviral agents, supportive therapy and trials of alternative medicines in many regions of the world. Given the fact that the liver is involved in the metabolism of many drugs, including nucleoside analogs and protease inhibitors that are currently used to treat COVID-19, hepatotoxicity from these drugs can arise.

A recent randomized controlled trial of lopinavir and ritonavir in severe COVID-19 reported that elevated levels of AST, ALT and total bilirubin occurred as adverse effects in a few patients.^41^ Another case series from Wuhan reported that 55.4% of patients experienced liver injuries after treatment with lopinavir and ritonavir.^26^ Fan et al. published a retrospective study on COVID-19 and observed that the utilization rate of this drug combination was significantly higher in patients with abnormal LFTs compared with patients without LFT elevations (56.1% *vs*. 25%, *p* = 0.009). In this study, 47.3% of the discharged patients showed elevated LFTs at baseline, and 23.7% developed abnormalities during hospitalization, suggesting emerging liver injury from drugs or during the course of the infection. Importantly, LFT elevation during the hospital stay was associated with prolonged length of hospitalization.^7^

Chloroquine, an old drug with a potential of repositioning for new treatment indications, has recently been tried in patients infected with SARS-CoV-2. After a profound success in inhibiting viral replication in vitro, concurrent clinical trials (>20) on chloroquine conducted at 10 hospitals across China have demonstrated superior efficacy in viral control.^42^ The pharmacodynamic activity of this drug in COVID-19 may involve the arresting of cytokine storms or the activation of CD8+ cells or by preventing endocytosis-mediated uptake of the virus.^43^ Importantly, hepatotoxicity related to chloroquine or hydroxychloroquine has rarely been reported.

In severe cases of COVID-19 with cytokine release, tocilizumab, an IL-6 antagonist, which is humanized IgG1 monoclonal antibody to the IL-6 receptor, has been used as a potential therapy for SARS-CoV-2. In previous clinical trials for other indications tocilizumab was reported to cause mild elevations of LFTs which were usually transient and commonly resolved within 2–6 weeks from exposure.^37^

Remdesivir is an experimental antiviral nucleotide analog with broad activity against coronaviruses^44^ that is currently being trialed for SARS-Cov-2 infection. Safety data from ongoing studies will guide on its use in patients, but so far no reports of liver toxicity have emerged.

## Patients with pre-existing liver disease

Scare data has been published for COVID-19 infection in patients with pre-existing liver disease. Experience from previous episodes of coronavirus infection can guide on the extent of hepatic involvement and on the management of patients with pre-existing liver disease. In SARS, the highest mortality rates were observed in the elderly and adults with underlying liver disease.^45^ Therefore, it has to be expected that the patients with COVID-19 are also more vulnerable to hepatic injury.^9^ In a case series from the Zhejiang province, a prevalence of 11% of underlying liver disease was reported. About half of them experienced symptoms for more than 10 days after the illness onset.^21^ In another study from Wuhan, 9% of patients had the underlying liver disease of cirrhosis or hepatitis.^23^ Li et al., who investigated risk factors involved with hepatic injury, stated that two patients had presented with alcoholic liver disease at baseline. One of them had a moderate elevation of ALT (120 U/L) within a week of hospitalization, while the other showed no such abnormalities.^11^ In the initial cohort described from China, 2.7% exhibited hepatitis B virus infection with no mention of worsening outcomes.^26^ Therefore, the association of the pre-existing liver conditions with disease prognosis and outcomes in COVID-19 will have to be evaluated by comprehensive data registries which recently started enrolling patients (e.g. COVID-Hep Registry and SECURE-Cirrhosis Registry).

### Liver transplant recipients

Management of post liver transplant recipients during the COVID-19 pandemic presents a special challenge for clinicians because of the limited data available and the crucial need to continue immunosuppressive drugs in these patients, which puts them at risk for more severe courses of COVID-19 infection and possible prolonged viral shedding. Case reports from China did not reveal an increased mortality in organ transplant recipients. Qin et al. reported the first case of SARS-CoV-2 infection in a patient with hepatocellular carcinoma who underwent liver transplantation.^29^ Lowering immunosuppression to the most acceptable level appears reasonable in infected liver transplant patients, in particular, in the setting of lymphopenia or clinical worsening of infection.^46^

In addition clinicians have to be aware of drug–drug interactions in the transplant setting. In particular immunosuppressive drugs and ritonavir-boosted antiviral therapies exhibit relevant interactions through CYP34A which lead to increased levels of calcineurin and mTOR inhibitors. Accordingly, chloroquine-based regimes or remdesivir (compassionate use program only) appear to be safe, while boosted protease inhibitors should be avoided (see Table 2). Additionally, preventive strategies in those vulnerable patients include early and prolonged screening with polymerase chain reaction-based testing for patients with early symptoms, a contact history or infection. Personal protective equipment in high risk settings can help to protect this vulnerable patient group.

**Table 2.** Drug–drug Interactions of experimental COVID-19 agents and immunosuppressive therapy.

Combination		Potential risk of interactions	Recommendations
Immunosuppressants	COVID-19 therapy		
Calcineurin inhibitor (tacrolimus or ciclosporin)	Atazanavir or lopinavir/ritonavir or chloroquine or hydrocholoquine	Potentially increased exposure of immunosuppressant	Dose adjustment or close monitoring
Sirolimus	Atazanavir or lopinavir/ritonavir	Potentially increased exposure of immunosuppressant	Avoid coadministration
Sirolimus	Chloroquine or hydrocholoquine	Potentially increased exposure of immunosuppressant	Dose adjustment or close. monitoring
Tacrolimus or ciclosporin or sirolimus	Tocilizumab	Potentially decreased exposure of immunosuppressant	Interaction of weak intensity; additional action/monitoring or dose adjustment unlikely required
Mycophenolate	Lopinavir/ritonavir	Potentially increased or decreased exposure of mycophenolate	Dose adjustment or close monitoring
Basiliximab	Tocilizumab	Enhanced immunosuppressive effect	Avoid coadministration
Azathioprine	Ribavarin	Myelotoxicity due to accumulation of 6-methylthioinosine monophosphate	Dose adjustment or close monitoring
Azathioprine	Tocilizumab or interferon-β	Additive hematological toxicity	Caution required; close monitoring of hematological parameters

Modified from Liverpool Drug interactions Group (5 April 2020; https://www.hep-druginteractions.org/).

## Summary and clinical recommendations

Liver function abnormalities – predominantly AST elevation – in COVID-19 appear to be frequent but not severe in most cases. Direct viral hepatotoxicity, DILI, ‘bystander effects’ during a systemic viral infection and potentially sepsis, or exacerbation of an underlying liver disease have to be considered. Ex vivo studies offer that SARS-CoV-2 can selectively target the liver, in particular cholangiocytes through ACE2, and thus hepatobiliary injury appears plausible. Irrespective of the mechanisms involved in the hepatic injury of patients with COVID-19, activation of the immune-mediated pathway seems to be critical. Special high risk populations require close monitoring. These include the elderly population, patients with end-stage liver disease and liver transplant recipients. Symptomatic treatment with acetaminophen and avoidance of non-steroidal anti-inflammatory drugs in cirrhosis is recommended. Cautious use of antiviral agents in patients with decompensated liver disease and drug–drug interactions in post liver transplant patients has to be considered. As emphasized by a recent position paper of the European Study of Liver Disease, elective procedures and routine tests should be postponed according to the risk–benefit at the given time. On the other hand, emergency medical care needs to be done with appropriate measures to prevent infection.^46^

## Supplemental Material

sj-pdf-1-ueg-10.1177_2050640620924157 - Supplemental material for Liver injury in COVID-19: The current evidence Click here for additional data file. Supplemental material, sj-pdf-1-ueg-10.1177_2050640620924157 for Liver injury in COVID-19: The current evidence by Saleh A Alqahtani and Jörn M Schattenberg in United European Gastroenterology Journal

## Figures and Tables

**Table 1.** Abnormalities in hepatobiliary function and inflammatory markers along with the proposed theories of hepatic injury in COVID-19.

Ref. no.	Author (et al.)	Publication type	Publication date	Study type	No. of patients with COVID-19	Pre-existing liver diseases	Hepatobiliary function markers	Inflammatory markers (and other relevant blood tests)	Proposed possible theories of hepatic injury
12	Huang C	Published	24 Jan 2020	Prospective case series	41	Chronic liver disease in one patient	AST (max., 48.0 U/L) increased in 37%, more in the ICU group	73% had LDH >245 U/L (max., 408 U/L)37% had LYM ≥1.0 × 10^9^/L (max., 1.1 × 10^9^/L)	Overall disease exacerbation: Cytokine storm
17	Chen N	Published	30 Jan 2020	Retrospective case series	99	No histories of hepatic diseases reported	ALT, AST, and TIBIL increased in 28%, 35%, and 18% of patients	CRP, ESR, IL-6, and LDH elevated in 86%, 85%, 52%, and 76% of patientsALB and LYM reduced in 98% and 35% cases, respectively	Overall disease exacerbation: Damage to T lymphocytes
22	Wang D	Published	7 Feb 2020	Retrospective case series	138	Chronic liver disease in 2.9% of patients	No significant liver abnormalities	LYM (Median: 0.8 × 10^9^/L) reduced in 70.3% cases, and LDH (Median: 261 U/L) increased in 39.9% of patients	Overall disease exacerbation: Cytokine storm induced by virus invasionSustained inflammatory response
26	Cai Q	Preprint	19 Feb 2020	Retrospective case series	298	2.7% had liver disease (details unspecified) Severe cases were associated with underlying diseases	14.8% experienced liver injury (ALT (max., 59.5 U/L) and AST (max., 65 U/L): 8.7 %, respectively)	CRP (max., 47.13 mg/dL) increased in 70% casesIL-6 (max., 28.72 ng/L) increased in 76% of patientsESR (max., 50 mm/h) increased in 60.9%LYM (min, 0.91 × 10^9^/L) reduced in 38.3%	Overall disease exacerbation: Inflammatory factor storm
21	Xu XW	Published	19 Feb 2020	Retrospective case series	62	11% had underlying liver disease (details unspecified) About half of them experienced symptoms for more than 10 days after illness onset	AST (max., 32 U/L) increased in 16% of patients	42% showed LYM reduction27% had LDH >245 U/LQuinolones and β-lactams administered if CRP >30mg/L	None described
18	Yang X	Ahead of Print	24 Feb 2020	Retrospective case series	52	No histories of hepatic diseases reported	29% had liver dysfunction (no specifics given)	LYM reduced in 85% cases	None described
23	Shi H	Published	24 Feb 2020	Retrospective case series	81	Hepatitis or liver cirrhosis in 9% of cases	AST (>40 U/L) increased in 53% of patients, lower in asymptomatic patients	LYM (≥1.0 × 10^9^/L) increased in 67%.CRP (Mean: 6.9 mg/L) was lower in asymptomatic patients	None described
27	Cao W	Preprint	25 Feb 2020	Retrospective case series	128	None described	ALT (Mean: 43.87 IU/L) and AST (Mean: 44.13 IU/L) increased in severe cases	LYM (Mean: 0.67 × 10^9^/L) reduced and CRP (Mean: 37.92 mg/L) increased in severe cases	None described
19	Zhang B	Preprint	27 Feb 2020	Retrospective case series with the data of non-survivors	82	Liver disease in 2.4% cases	ALT (>40 U/L), AST (>40 U/L), and TBIL (>20.5 mmol/L) increased in 30.6%, 61.1%, and 30.6% cases	LYM (<1.0 × 10^9^/L), ALB (<40 g/L) and CD8+ cells (<220 × 10^9^/L) reduced in 89.2%, 77.8%, and 98.3% cases, respectivelyLDH (>250 U/L) and CRP (>10 U/L) elevated in 93.2% and 100% of patients, respectively	Overall disease exacerbation: Viral invasion of organsInflammatory factor elicitationPerturbation of immune system
25	Guan WJ	Ahead of print	28 Feb 2020	Retrospective case series	1099	Hepatitis B in 2.1% of patients	Increase of AST (>40 U/L) in 22.2%, ALT (>40 U/L) in 21.3%, and TBIL (>17.1µmol/L) in 10.5%	Elevation of CRP (≥10 mg/L) and LDH (≥250 U/L) in 60.7% and 41.0%, respectively	None described
7	Fan Z	Preprint	28 Feb 2020	Retrospective case series	148	None described	50.7% of patients had liver function abnormalities at admission21.6%, 18.2%, 17.6%, 6.1%, and 4.1% patients had elevated AST, ALT, GGT, TBIL, and ALP, respectively.	35.1% showed LDH elevationCD4+ and CD8+ T cells decreased and CRP increased in abnormal liver function group	Drug-induced inflammatory factor stormViral infection of the liver
8	Zhang C	Published	4 Mar 2020	Review with a description of unpublished case series	56	3.6% of patients had pre-existing liver diseases (details unspecified)	28.6% of cases had abnormal liver functionsOne fatal case with evaluated liver injury (ref. no. 3)	None described	Direct viral infection of liver cellsDrug-induced immune dysfunction
24	Huang Y	Preprint	5 Mar 2020	Retrospective case series with the data of non-survivors	36	No histories of hepatic diseases reported	ALT, AST and TBIL increased in 13.3%, 58.1%, and 12.9% of patients, respectively	LYM and ALB decreased in 70.6% and 80.6% cases, respectively. LDH and IL-6 increased in all patients. CRP increased in 96.97% of cases	None described
11	Li L	Preprint	10 Mar 2020	Retrospective case series	85	Hepatitis B, alcoholic liver disease, and fatty liver disease (*n*=2 in each category)	24.7% had ALT elevation at admission	CRP ≥20mg/L and LYM count <1.1 × 10^9^/L were independent risk factors for hepatic injury. ALB (Mean: 33.4 g/L) in ALT elevated group was significantly lower	Inflammatory cytokine stormDeterioration of the disease with a dynamic process
13	Cui Y	Ahead of print	17 Mar 2020	Case report of an infant	1	A healthy infant with no medical history	ALT (84 IU/L), AST (100 IU/L) and TBIL (33.7 µmol/L) elevated	CD8+ T cells (2208 cells/µL) and LYM count (5.22 × 10^9^/L) elevated	Overall disease exacerbation: An initial increase in T helper 2 cell response, followed by suppression of inflammatory responses
9	Xu L	Ahead of print	14 Mar 2020	Review with a comment of unpublished data	Not described	Not described	GGT increased in severe cases (values not reported)	Not described	Direct virus-induced cytopathic effectsOvershooting of inflammatory responsesViral hepatitis Drug-induced
28	Bangash MN	Ahead of Print	20 Mar 2020	Review	–	–	–	–	Virally induced cytotoxic T cellsInduction of a dysregulated innate immune response
29	Qin J	Ahead ofprint	27 Mar 2020	Case report	1	Hepatitis BHCC	ALT (80 U/L) and AST (132 U/L) declined post-liver transplantation but elevated gradually	LYM reduced to 0.64 × 10^9^/L	None described
30	Xie H	Ahead of print	2 Apr 2020	Retrospective case series	79	Patients with previous liver diseases were excluded	31.6%, 35.4% and 5.1% of patients had elevated ALT, AST and TBIL, respectively. Median (range) values were 36.5 (17.5–71.5) U/L, 34.5 (25.3-–55.3) U/L and 12.7 (8.1–15.4) mmol/L, respectively.	CRP (max., 79.6 µmol/L) and ESR (max., 58 mm/h) increased; while LYM reduced (min., 0.9 × 10^9^/L)	Overall disease exacerbation: Disease severity
31	Zhang Y	Ahead of print	2 Apr 2020	Retrospective case series	115	Two patients had chronic Hepatitis B (excluded)	ALT and AST increased in 9.57% and 14.78% patients, respectively on admission. TBIL elevation was rarely observed. Mean levels higher in severe cases.	54.78% had reduced ALB, significantly lower in severe cases. 57.39% had increased CRP, higher in severe cases (80.75±69.18). LDH level (Mean±SD:346.10±257.26) significantly elevated in severe cases	Dysfunction of immune system
32	Ding Q	Ahead of print	20 Mar 2020	Prospective case series	Five patients who had both COVID-19 and influenza infection	Two patients had Hepatitis B	ALT and AST increased in 40% (each) of cases, respectivelyAcute liver injury occurred in 60% of patients	CRP increased in 80%, while LYM reduced in 80% of cases.	None described
33	Zhao D	Ahead of print	12 Mar 2020	Prospective case series	19	Hepatitis B in one patient	Liver function damage is more frequent in COVID-19 than other pneumonia patients. 27.78% (each) cases had elevated ALT and AST, respectively, while GGT increased in 44.4%	LDH increased in 31.58% of cases	Overall disease exacerbation: Dysregulation of immune system
34	Feng G	Published	30 Mar 2020	Review article	–	–	–	–	Hypoxia-reperfusion dysfunctionSystemic inflammation responseDrug-induced Viral infection of the liverACE2 positive cholangiocytes
35	Chen H	Published	12 Feb 2020	Retrospective case series	9	None described	33% each had increased ALT and AST, respectively. One had ALT reaching 2093 U/L and AST reaching 1263 U/L	56% patients had LYM (<1.0 × 10^9^ cells/L) reduction75% of cases had elevated CRP (>10 mg/L)	None described
36	Pan F	Ahead of print	13 Feb 2020	Retrospective case series	21	None described	Elevated levels of ALT (max., 107 U/L) and AST (max., 95 U/L) were observed	Elevated CRP (max., 88.6 mg/L), LDH (max., 377 U/L) and ESR (max., 93 s) were reported	None described

ALB: albumin; ALP, Alkaline phosphatase; ALT: alanine aminotransferase; AST: aspartate aminotransferase; CRP: C-reactive protein; ESR: erythrocyte sedimentation rate; GGT: gamma-glutamyl transferase; HCC: hepatocellular carcinoma; ICU: intensive care unit; IL-6: interleukin-6; LDH: lactate dehydrogenase; LYM: lymphocytes; max.: maximum; min.: minimum; TBIL: total bilirubin
