# Supplementary material for: Liver injury in COVID-19: The current evidence
Source: United European Gastroenterol J. 2020 May 26;8(5):509–19. doi: 10.1177/2050640620924157 (PMC7268949; doi:10.1177/2050640620924157)
Supplement: sj-pdf-1-ueg-10.1177_2050640620924157 - Supplemental material for Liver injury in COVID-19: The current evidence [file sj-pdf-1-ueg-10.1177_2050640620924157.pdf]

Supplementary Information- Liver Injury in COVID-19: the current evidence

Saleh A. Alqahtani [1,2] and Jörn M. Schattenberg[3]

1. Liver Transplantation Unit, King Faisal Specialist Hospital & Research Center, Riyadh, Saudi Arabia, 2. Division of Gastroenterology and Hepatology, Johns Hopkins University, Baltimore, MD, USA. 3. Metabolic Liver Research Program, I. Department of Medicine, University Medical Center, Mainz, Germany

Search Strategy

A literature search was performed as advanced Search on Google Scholar as of May 22nd and included the keyword: “COVID”, “nCoV”, “novel Corona”, “SARS-CoV-2” added with the following words: “gastrointestinal”, “diarrhea”, “vomiting”, “anorexia”, “abdominal”, “hepatic”, “liver”, “hepatobiliary” yielding 9 relevant articles. A further manual search of literature from the references and citations of those 9 articles has provided 46 more manuscripts, of which, 13 were directly related to the gastrointestinal route of COVID-19 transmission or complications due to the viral infection, and 7 provide clinical/pathological evidence on the hepatic complications due to this specific infection. The rest of the literature (n=26) provided evidence on general clinical characteristics, some including hepatobiliary data. In addition an advanced search of PubMed database using the selected keywords yielded 5 articles that were also identified in Google scholar search. Using the “Similar Articles” option in the PubMed platform this yielded articles (n=50) which provide evidence on general clinical characteristics.
